# Supplementary material for: PKCɛ switches Aurora B specificity to exit the abscission checkpoint
Source: Nat Commun. 2016 Dec 22;7:13853. doi: 10.1038/ncomms13853 (PMC5192180; doi:10.1038/ncomms13853)
Supplement: Supplementary Information — Supplementary Figures [file ncomms13853-s1.pdf]

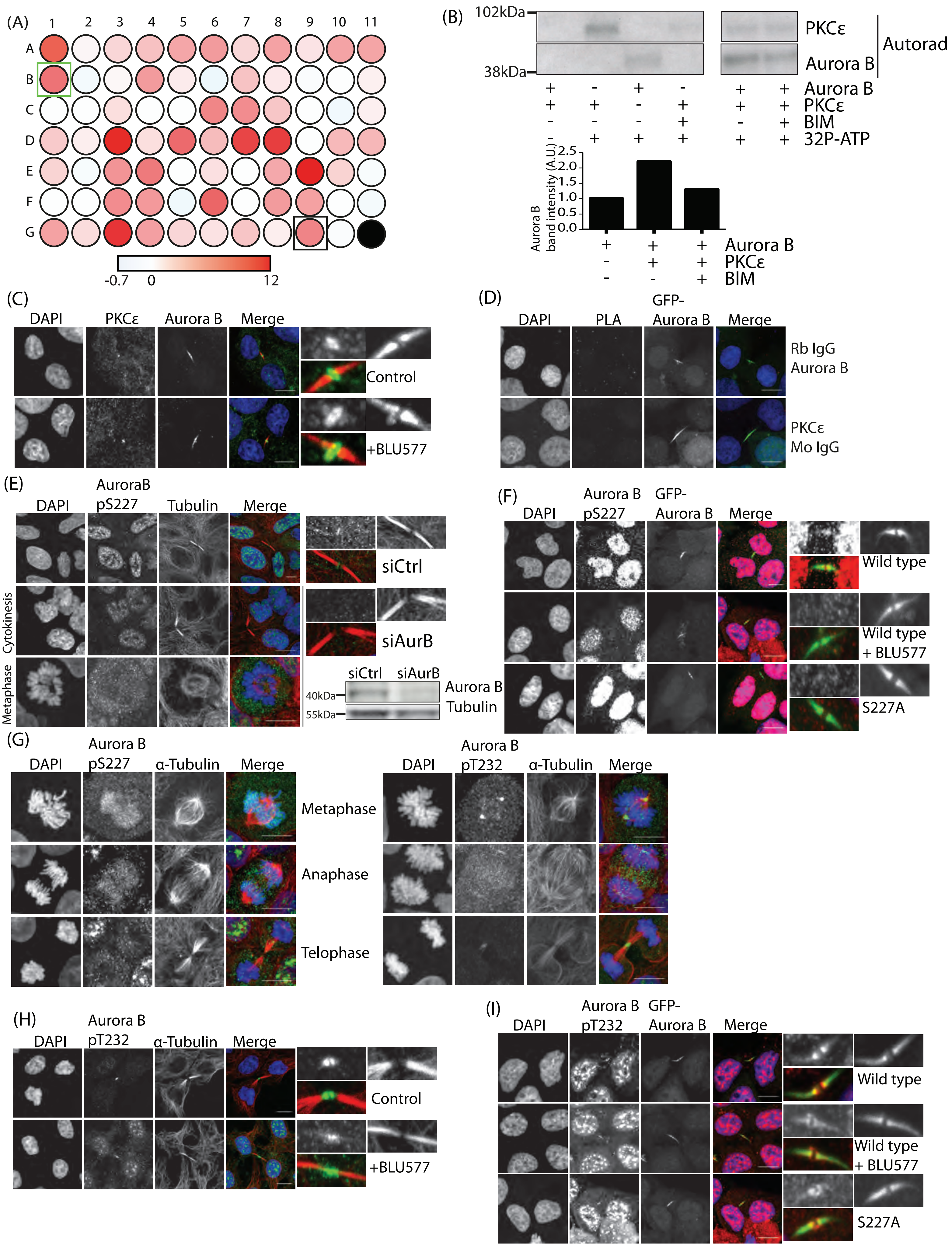

Supplementary Figure 1: PKCε phosphorylates Aurora B in vitro and in vivo

(A) Heatmap representing PKCε substrate peptide array. PKCε pseudeosubstrate positive control is at position G9, Aurora B S227 peptide is at position B1. Values for all peptides can be found in Supplementary Dataset 1. **(B)** In vitro kinase assay of PKCε and Aurora B with and without the PKC inhibitor BIM (1μM). Graphs shows analysis by densitometry. **(C)** Confocal images of DLD1 cell line demonstrating midbody localization of PKCε (green) and Aurora B (red). Inhibition of PKCε with BLU577 (500nM) had no effect on the localization of these proteins. Scale bar = 10μm. **(D)** Proximity ligation assay negative controls. Primary antibodies pairs of endogenous Aurora B (top panel) and non-specific rabbit IgG or PKCε (lower panel) and non-specific mouse IgG were used to demonstrate the specificity of the PLA reaction in Figure 1D. Scale bar = 10μm. **(E)** DLD1 cells were transiently transfected with siCtrl or siAurB for 24 hours before fixing and staining for phospho-S227 Aurora B (green) and tubulin (red). The nuclear staining with the phospho-S227 antibody is largely unaffected by siRNA knockdown of Aurora B, indicating a cross-reacting antigen. The signal is however lost from the midbody indicating that the antibody is specifically detecting S227 phosphorylated Aurora B. Scale bare = 10μm. Western blot after siRNA treatment shows loss of Aurora B protein (inset). **(F)** Confocal images of the DLD1 GFP-Aurora B cell lines (green) ± BLU577 (500nM, 30 min). Midbody localization of S227 phosphorylated Aurora B (red) is observed in the wild type expressing cells although this is sensitive to PKCε inhibition with BLU577. Detection of phospho-S227 is absent at the midbody in S227A expressing cells. Scale bar = 10μm. **(G)** Mitotic localization of S227 phosphorylated Aurora B (left panel) (green) and T232 phosphorylated Aurora B (right panel) (green). Microtubules are stained in red. Scale bar = 10μm. **(H)** Aurora B T232 phosphorylation (green) is insensitive to BLU577 (500nM) in the DLD1 parental cell line and **(I)** also in both the DLD1 GFP-Aurora B cell lines (green). Microtubules are stained in red. Scale bar = 10μm.

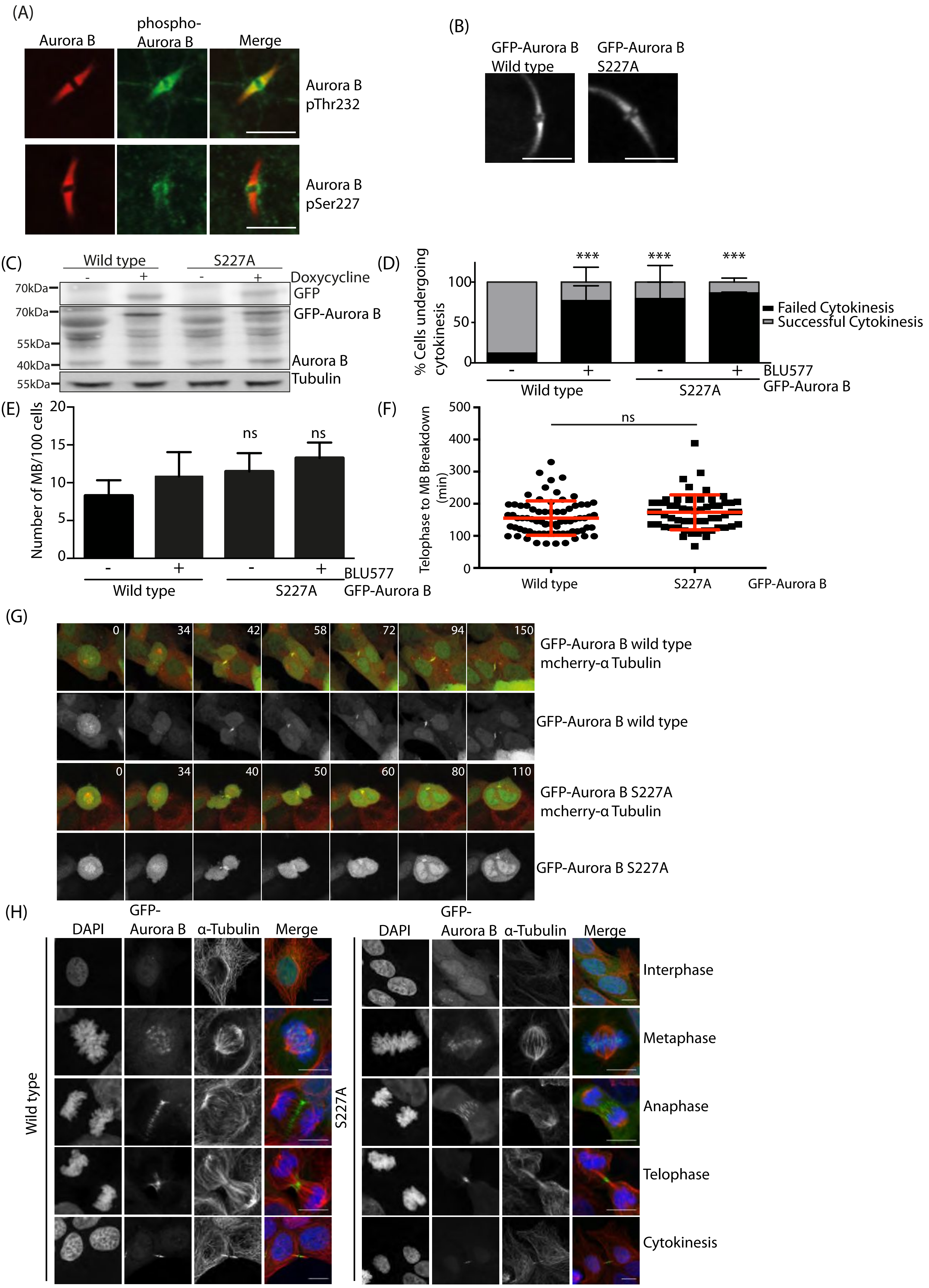

Supplementary Figure 2: Aurora B S227 phosphorylation is required for successful cytokinesis
(A) Confocal images of midbodies stained for phosphorylation of T232 or S227 (green) and Aurora B (red) . Scale bar represents 5µm. **(B)** Confocal images of GFP-Aurora B demonstrating midbody ring localization. Scale bar represents 5µm. **(C)** Representative western blot demonstrating induction of GFP-Aurora B WT and S227A after 24h doxycycline treatment (1µg per mL). **(D)** Flp-In Trex HEK293 cells expressing inducible GFP-Aurora B wild type and S227A were observed by live cell time-lapse microscopy for the outcome of cytokinesis as in Figure 2B. Graph represents the mean (± s.e.m.) of 3 independent experiments where a minimum of 100 cells were scored per condition. Two way ANOVA, *** = P≤0.001. **(E)** DLD1 GFP-Aurora B cell lines ± BLU577 were induced for 24h as in Figure 2A and assessed for the number of cells still linked by midbodies by confocal microscopy. A modest but not significant (P>0.05) increase was observed if PKCε was inhibited (8.27%±2.06 vs 10.83%±3.22) or Aurora B could not be phosphorylated on S227 (S227A 11.47%±2.43). The graph represents the mean ± s.e.m. of three independent experiments where a minimum of 500 cells was counted per experimental condition. Student's *t*-test, ns= P>0.05. **(F)** DLD1 GFP-Aurora B mCherry-α tubulin cell lines were scored for the timing of telophase onset to midbody breakdown (in both successful and failed cytokinesis) using live cell time-lapse microscopy (155.2±53.57min vs 173.3±54.24 min P=0.580). Graph represents 3 independent experiments (error bars equal the mean ± s.d.) and a minimum of 50 cells scored per condition. Student's *t*-test, ns= P>0.05. **(G)** Still images from live cell time-lapse microscopy of cells in Figure 2B. **(H)** Confocal images of DLD1 GFP-Aurora B expressing cells (green) in each phase of mitosis. Microtubules are visualised in red. Scale bar = 10µm.

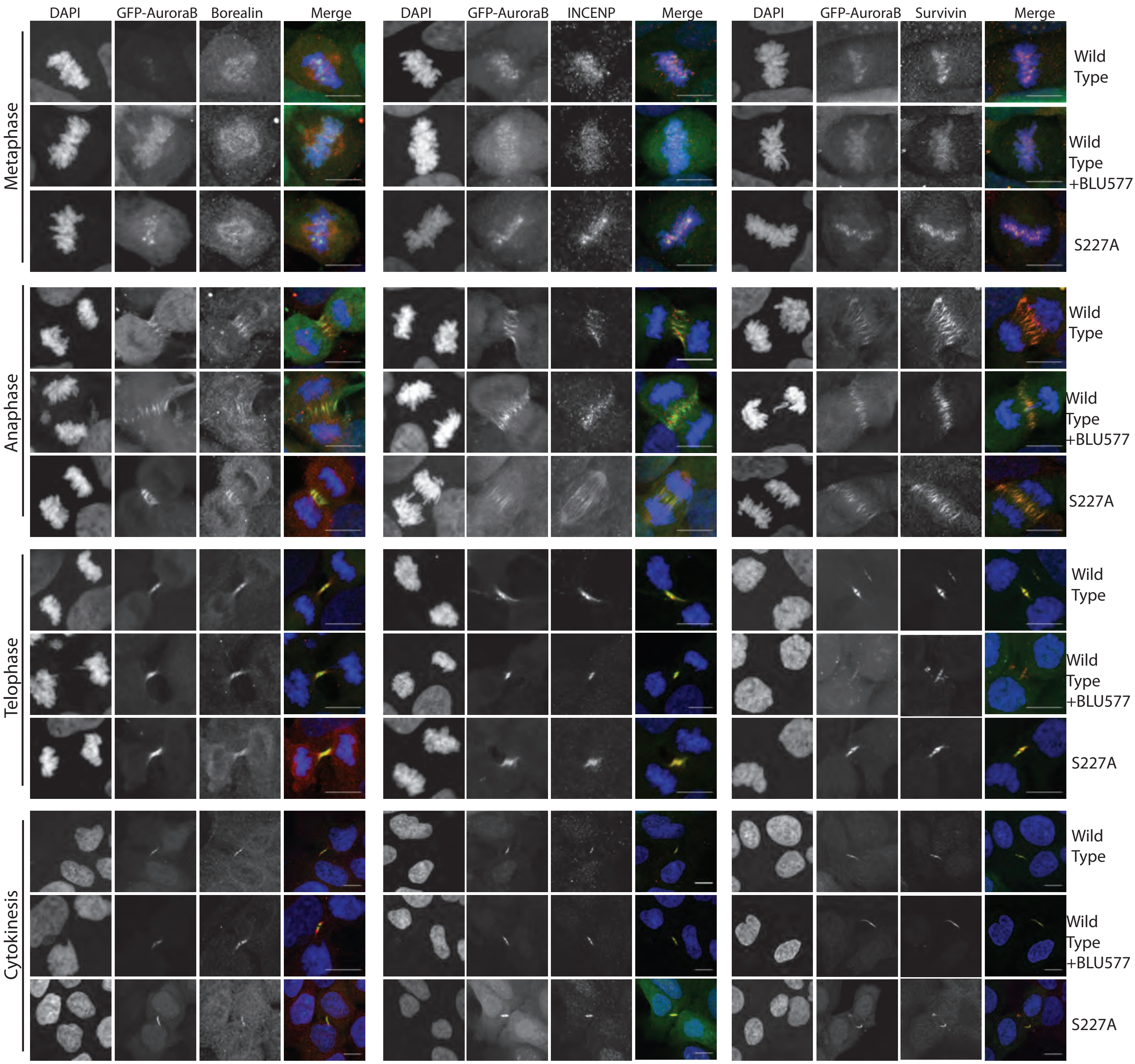

Supplementary Figure 3: Inhibiting PKCe dependent phosphorylation of Aurora B does not affect affect CPC localisation
Confocal images of DLD1 GFP-Aurora B cell lines (green) ± BLU577 demonstrating co-localisation of the CPC components Borealin, INCENP and survivin (red) at any stage of mitosis or cytokinesis. Scale bar = 10µm.

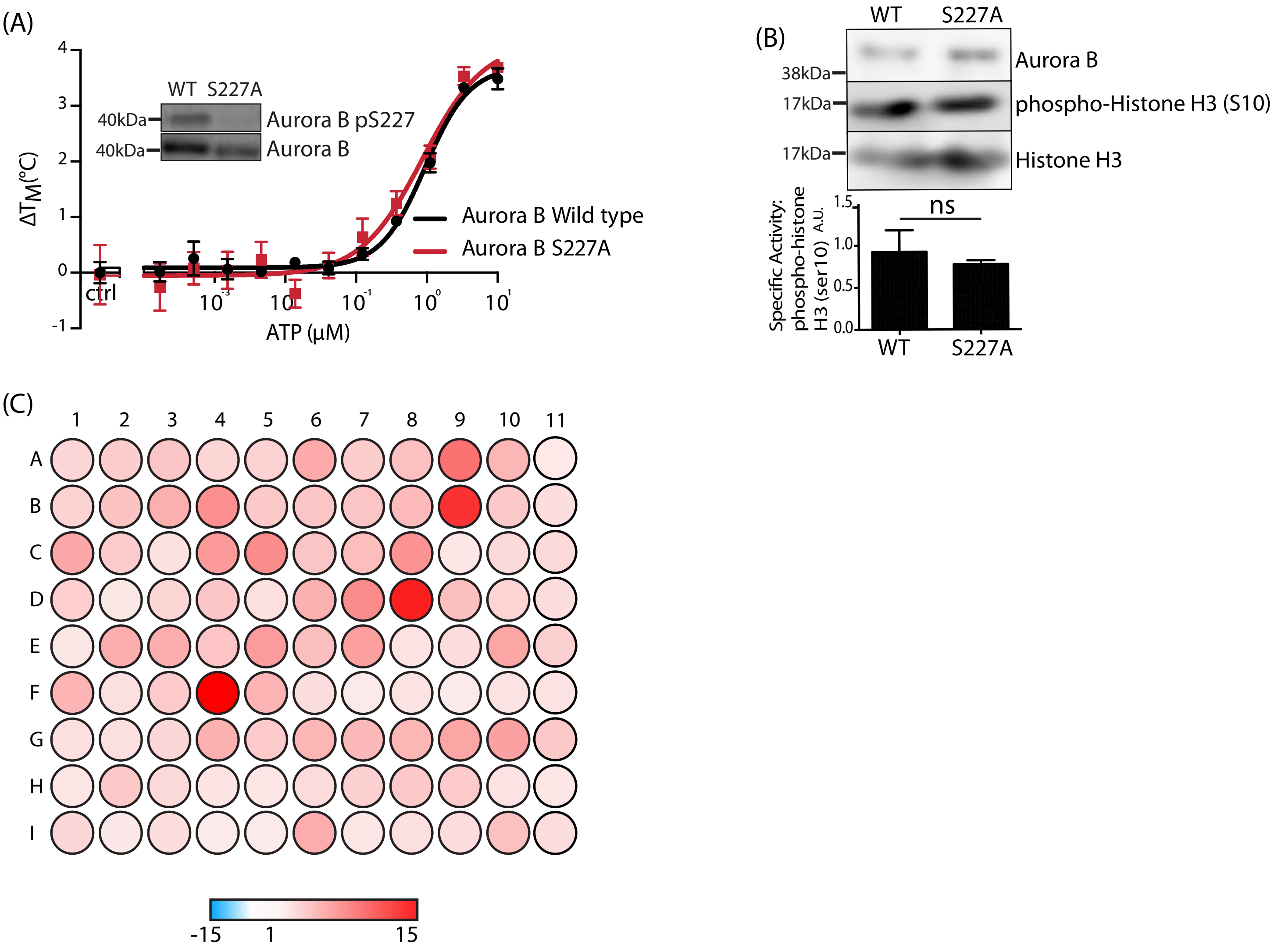

Supplementary Figure 4: Aurora B WT and S227A are both functionally active kinases

(A) Thermal shift assay of recombinant human Aurora B wild type or S227A with increasing concentration of ATP. Data is representative of the mean of one experiment (n=2). Recombinant proteins were immunoblotted for S227 phosphorylation (inset). **(B)** In vitro kinase assay of recombinant Aurora B WT and S227A using phosphorylation of Histone H3 at S10 as an output of phosphotransferase activity. Graph represents the mean (± s.e.m.) of 3 independent experiments normalized to the amount of recombinant Aurora B. Student's *t*-test, ns = P>0.05. **(C)** Heatmap representing Aurora B substrate array. Spots represent the ratio of Aurora B WT/Aurora B S227A. Values for each peptide can be found in Supplementary Dataset 2.

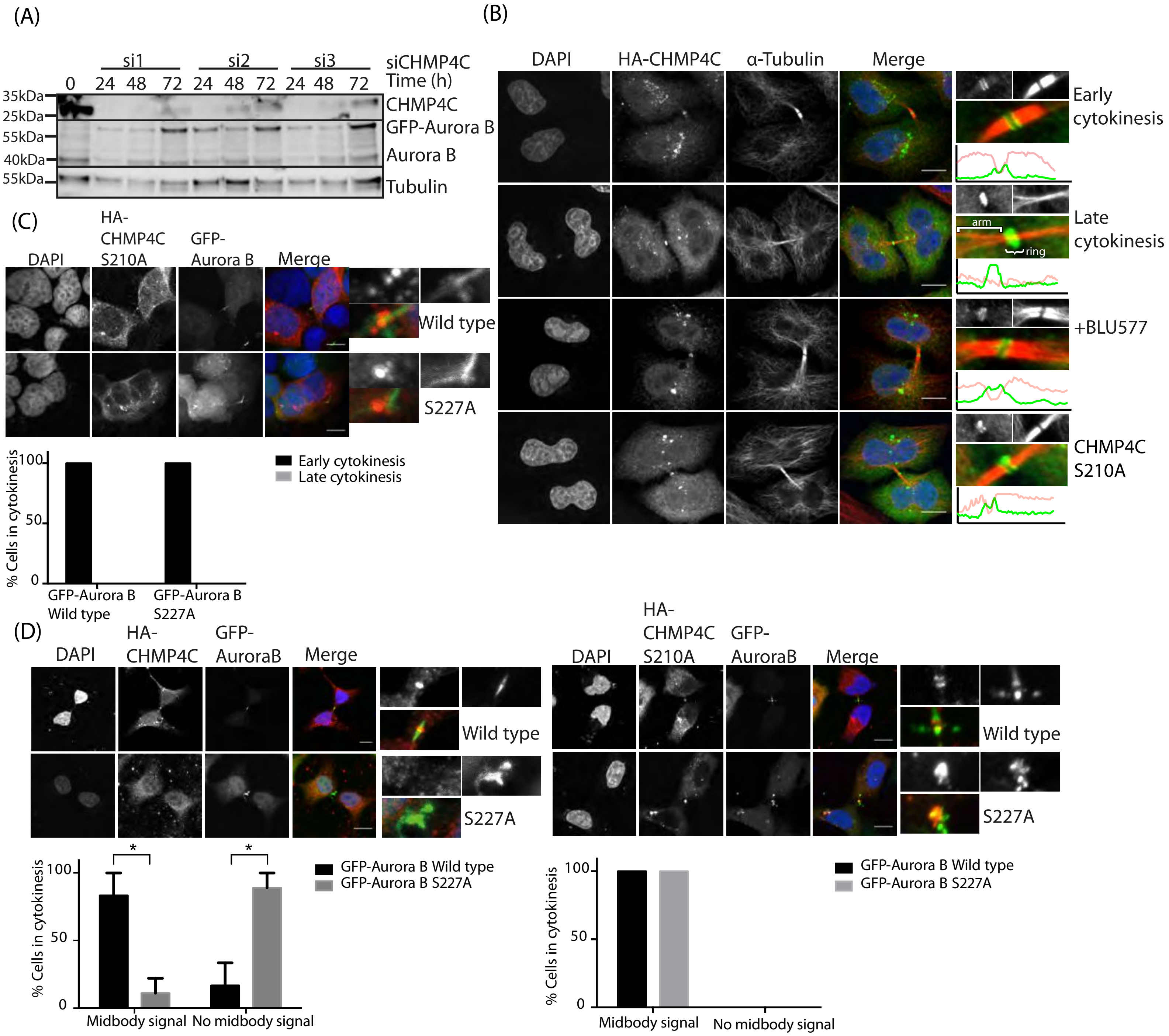

Supplementary Figure 5: Localization of CHMP4C to the midbody ring is required for completion of abscission

(A) Representative western blot from Figure 4A demonstrating CHMP4C knockdown using 3 independent siRNA and induction of GFP-Aurora B at the time points specified. **(B)** Confocal images of HeLa cells transiently transfected with HA-CHMP4C (green) to demonstrate early and late cytokinesis localization. Microtubules are stained in red. Early cytokinesis is defined by the localisation of CHMP4C to the midbody arms, whilst late cytokinesis is defined by CHMP4C localisation to the midbody ring as described by Carlton *et. al* (2012). Inset shows magnified view of midbodies and intensity profiles demonstrating the midbody localization of CHMP4C (green) and tubulin (red). Scale bar = 10μm. **(C)** Transient transfection of HA-CHMP4C S210A mutant (red) into DLD1 GFP-Aurora B WT and S227A cell line.(green) to observe midbody localization of both proteins. Cells were scored for the presence or absence of HA-CHMP4C at the midbody. Scale bar = 10μm. Student's *t*-test, ns= P>0.05. **(D)** HEK293 GFP-Aurora B cell lines transiently transfected with HA-CHMP4C (left panel) or HA-CHMP4C S210A mutant (rightpanel). GFP-Aurora B (green) and HA-CHMP4C (red). Cells were scored for the presence or absence of HA-CHMP4C at the midbody. A minimum of 12 high resolution, single cell images per condition from 4 experiments were acquired, a representative image is shown here. Scale bar = 10μm. Student's *t*-test, ns= P>0.05, * = P≤0.05.

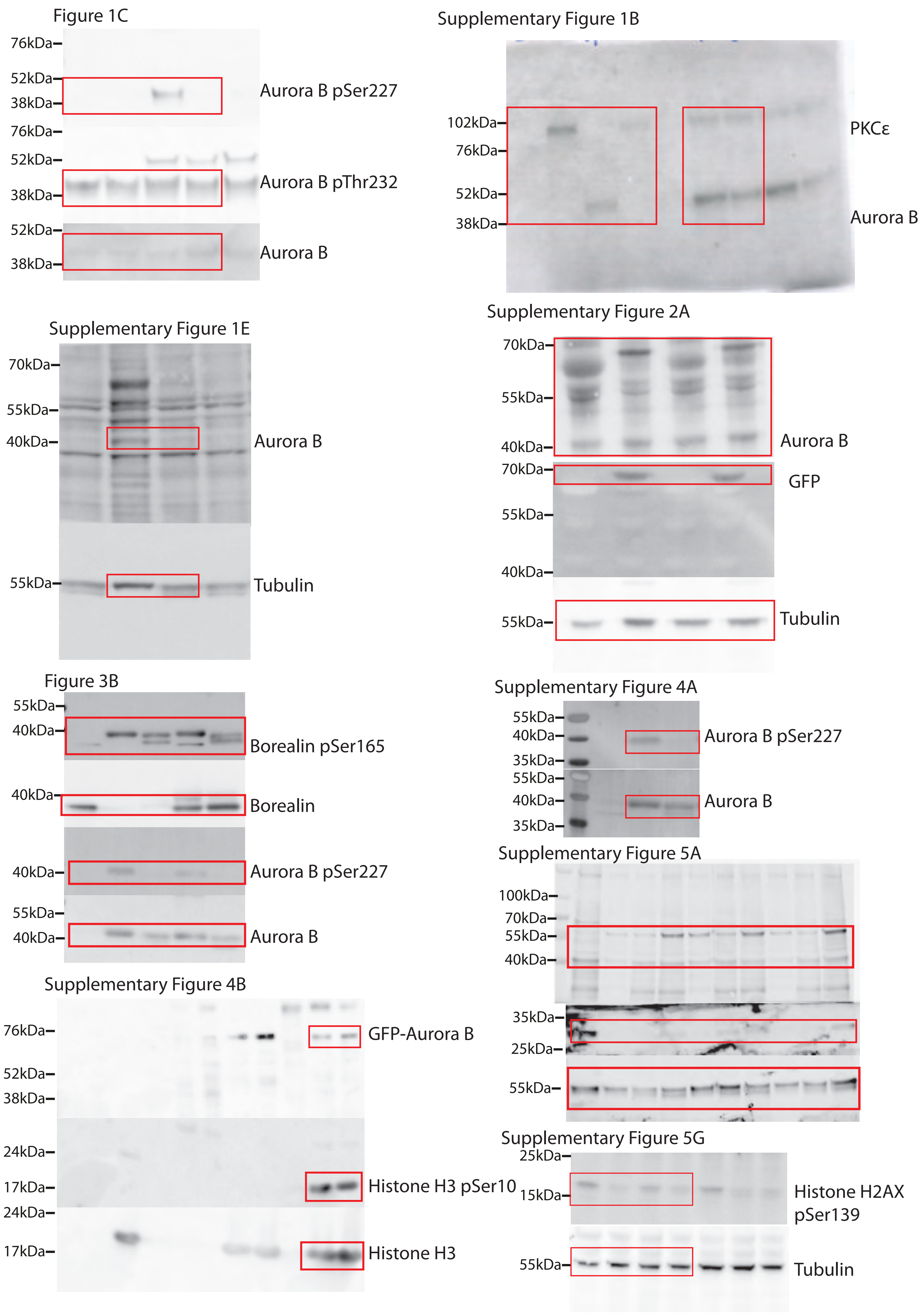

Supplementary Figure 6: Western Blots
Uncut western blots from main figures. Red boxes show portions of blots described.
